# Supplementary material for: Chromatin attachment to the nuclear matrix represses hypocotyl elongation in Arabidopsis thaliana
Source: Nat Commun. 2024 Feb 12;15:1286. doi: 10.1038/s41467-024-45577-5 (PMC10861482; doi:10.1038/s41467-024-45577-5)
Supplement: Supplementary file 1 — Supplementary Information [file 41467_2024_45577_MOESM1_ESM.pdf]

Supplementary Fig. 1

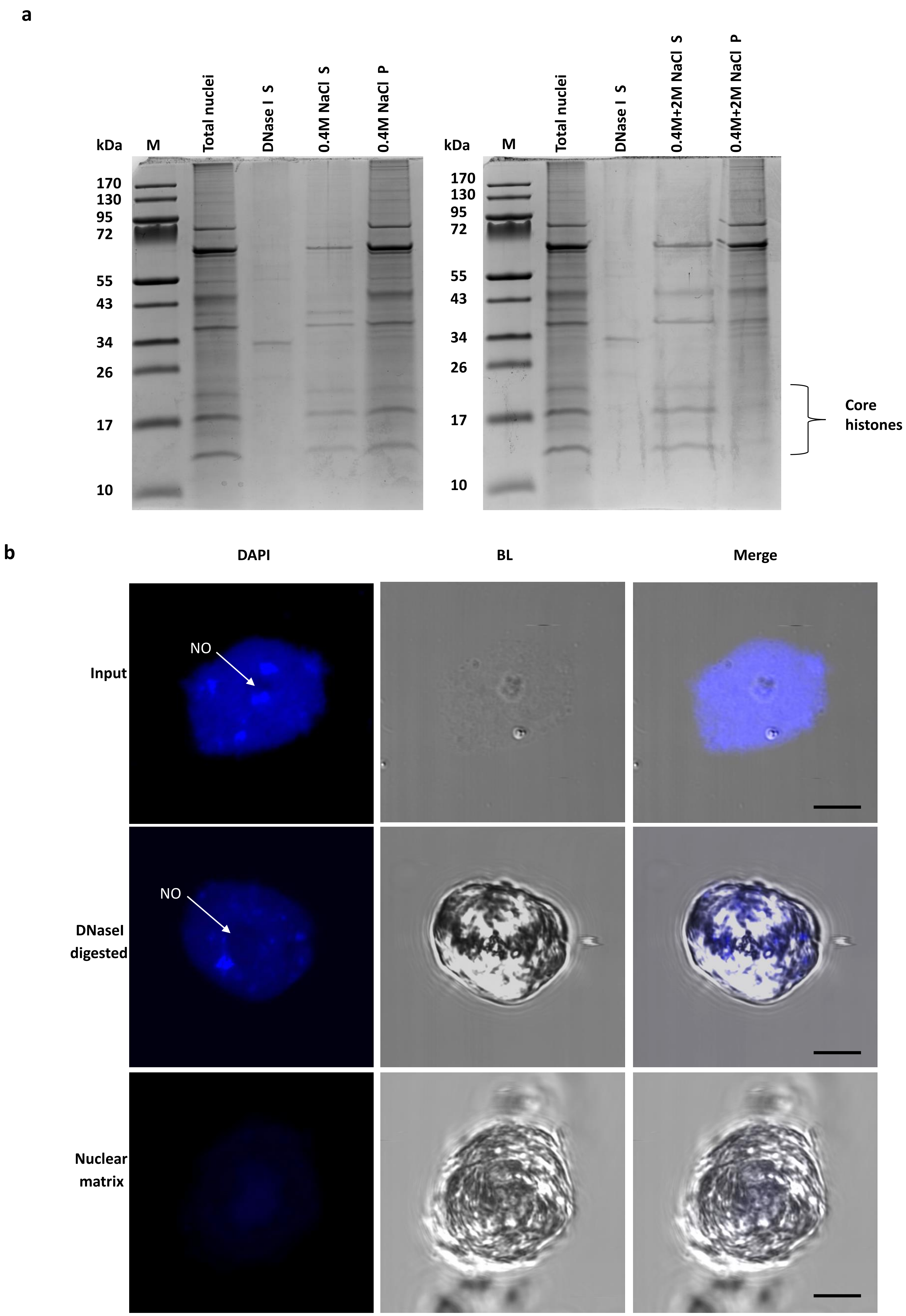

Supplementary Fig. 1. Validation of extracted protein and DNA from nuclear matrix.
(a). Coomassie-stained SDS-tricine gels of proteins during the extraction processes. M-Marker, S-Supernant, P-nuclei pellet .
(b). Confocal images and DAPI staining of nuclei pellet at three stages of nuclear matrix isolation. NO-nucleolus, Scale bar= 5 µm.

Supplementary Fig. 2

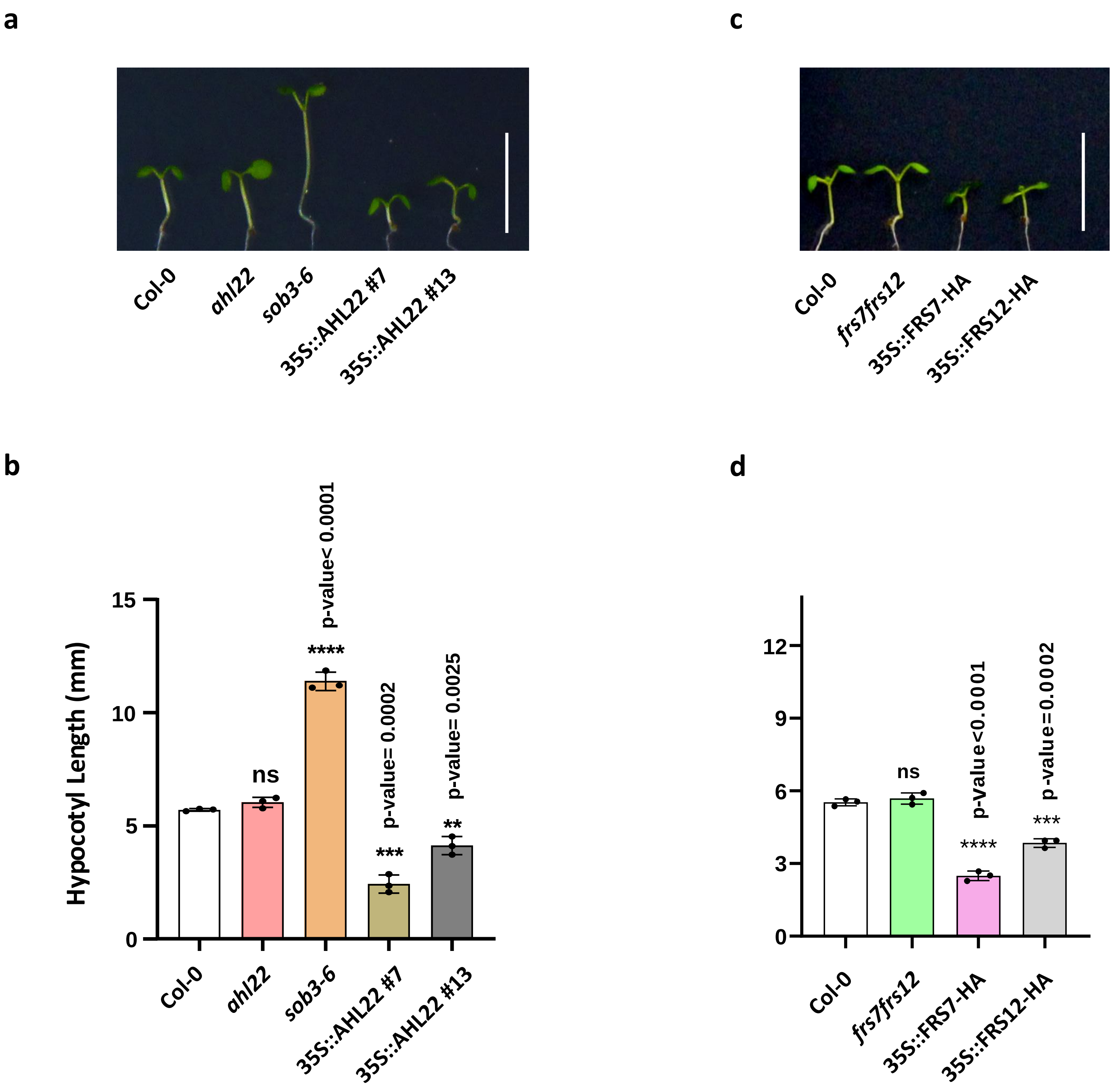

Supplementary Fig. 2. Hypocotyl phenotype

(a,c). The hypocotyl phenotype of indicated lines grown vertically under LD conditions (16h at 23 °C under 22 $\mu mol \cdot m^{-2} \cdot s^{-1}$ continuous white light, 8h at 23 °C dark) for 5 days. Scale bar= 1 cm.

(b,d). Hypocotyl lengths of indicated lines grown under LD conditions (16h at 23 °C under 22 $\mu mol \cdot m^{-2} \cdot s^{-1}$ continuous white light, 8h at 23 °C dark) for 5 days.

Average length of three independent measurements ± standard deviations are shown. Each measurement with n=30 plants , 3 replicates in total. Unpaired two-tailed Student's t-test was used to determine significance between wild type and mutants or overexpression lines. N.S. *p value* >0.05, **p value* ≤ 0.05, ***p value* ≤ 0.01, ****p value* ≤ = 0.001, *****p value* ≤ = 0.0001.

Supplementary Fig. 3

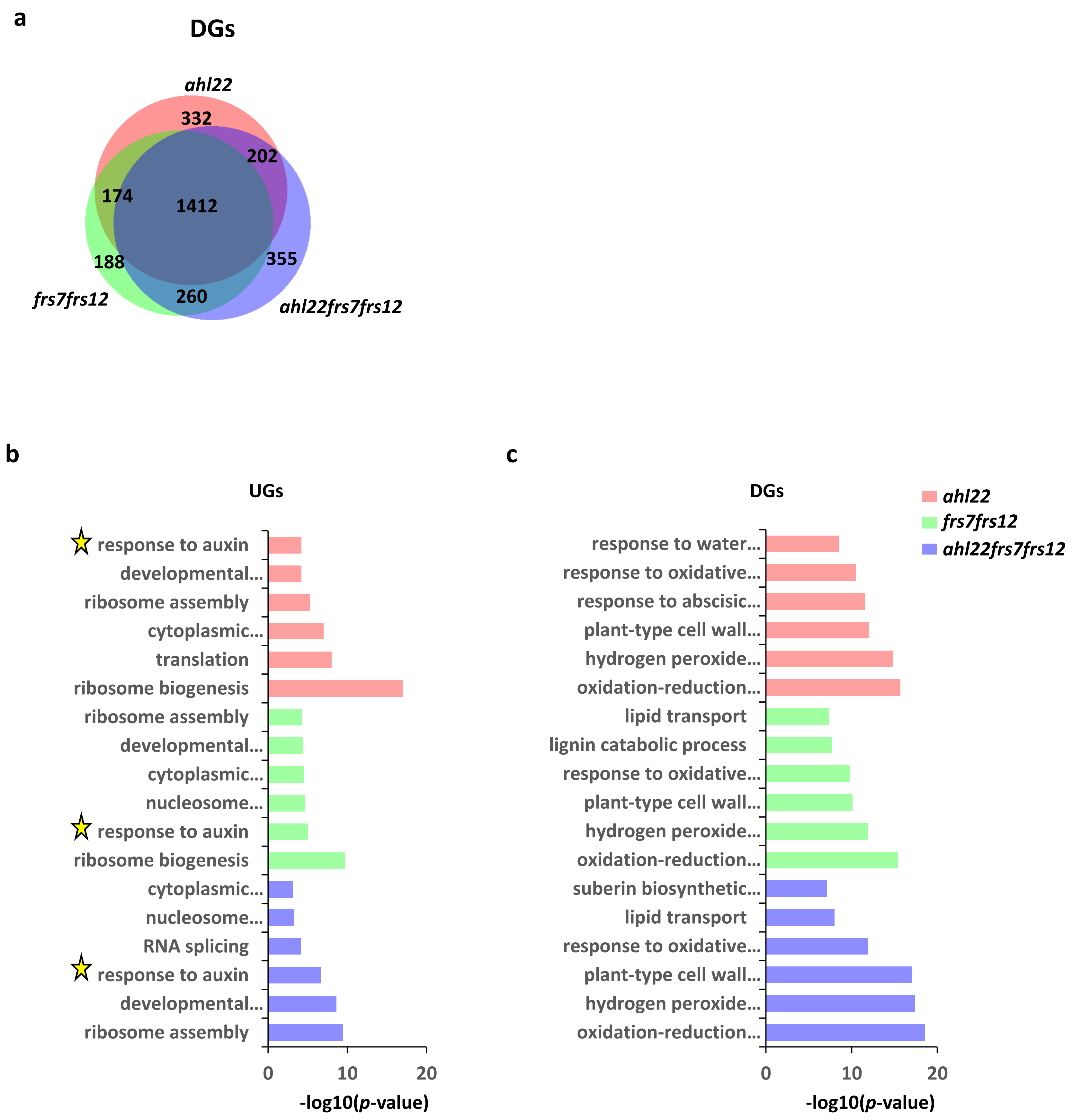

Supplementary Fig. 3. Transcriptomic analysis in the mutants. (a) Venn diagram showing the overlap of down-regulated gene in corresponding genotypes; enriched biological processes of significantly UGs (b) and DGs (c) in *ahl22, frs7 frs12* and *ahl22 frs7 frs12* compared to Col-0. The X-axis represent negative log10 (p-value). Yellow stars indicate pathway response to auxin.

Supplementary Fig. 4

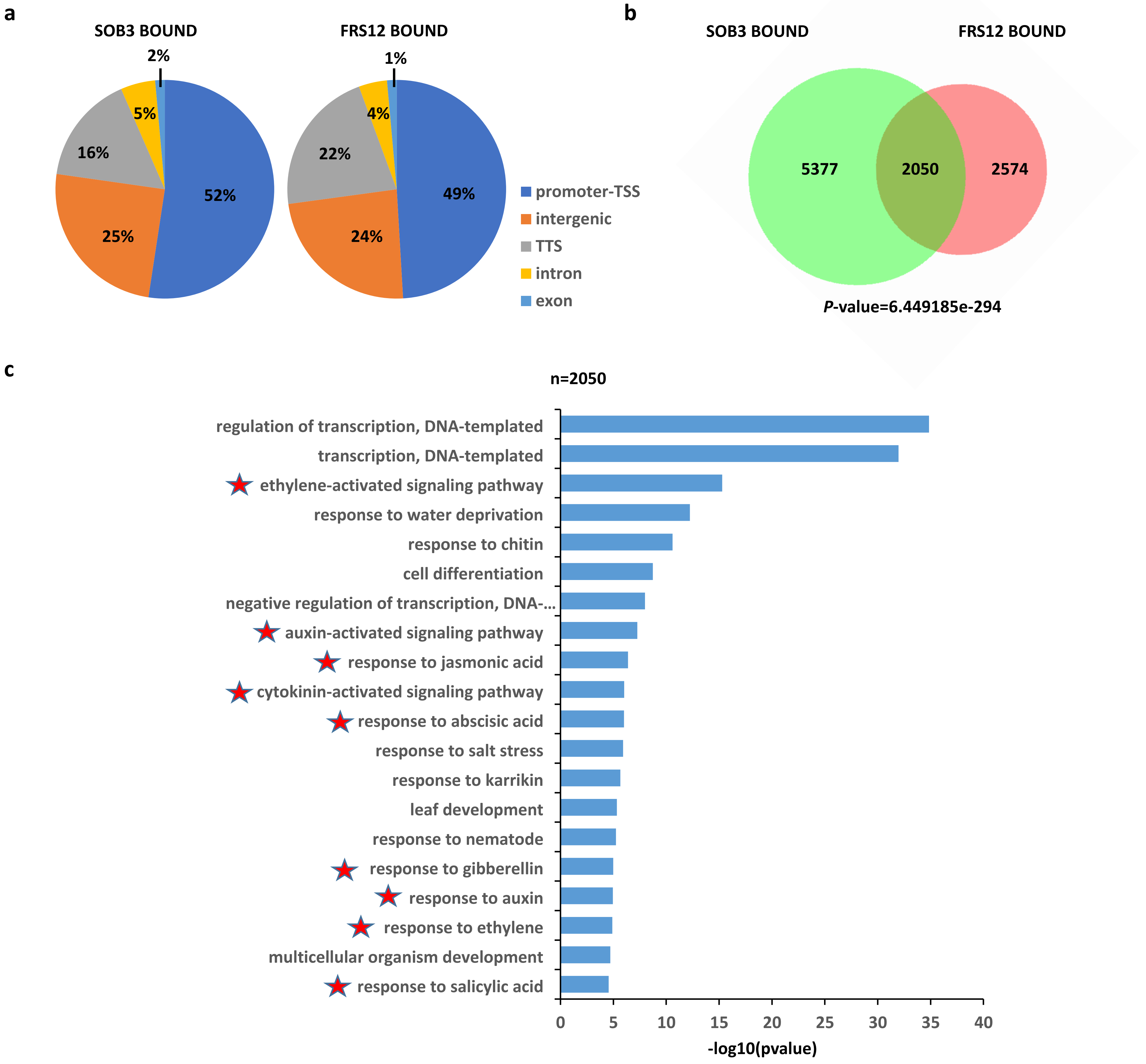

Supplementary Fig. 4. SOB3 (AHL29) and FRS7/12 co-target to phytohormone pathways.
(a). Distribution of published SOB3 and FRS12 binding sites determined from ChIP-seq data. ''Promoter-TSS'' is defined as -1,000 bp to +100 bp in relation to the transcription start site. ''TTS'' is defined as -100 bp to +1,000 bp in relation to the transcription termination site.
(b). Venn diagram showing overlap of SOB3 BOUND and FRS12 BOUND sites. Significance was tested using a hypergeometric test.
(c). Enriched biological processes of common targeted genes (n=2050) of SOB3 BOUND and FRS12 BOUND. The X-axis represent negative log10 (*p-value*). Red stars indicate phytohormone pathways.

Supplementary Fig. 5

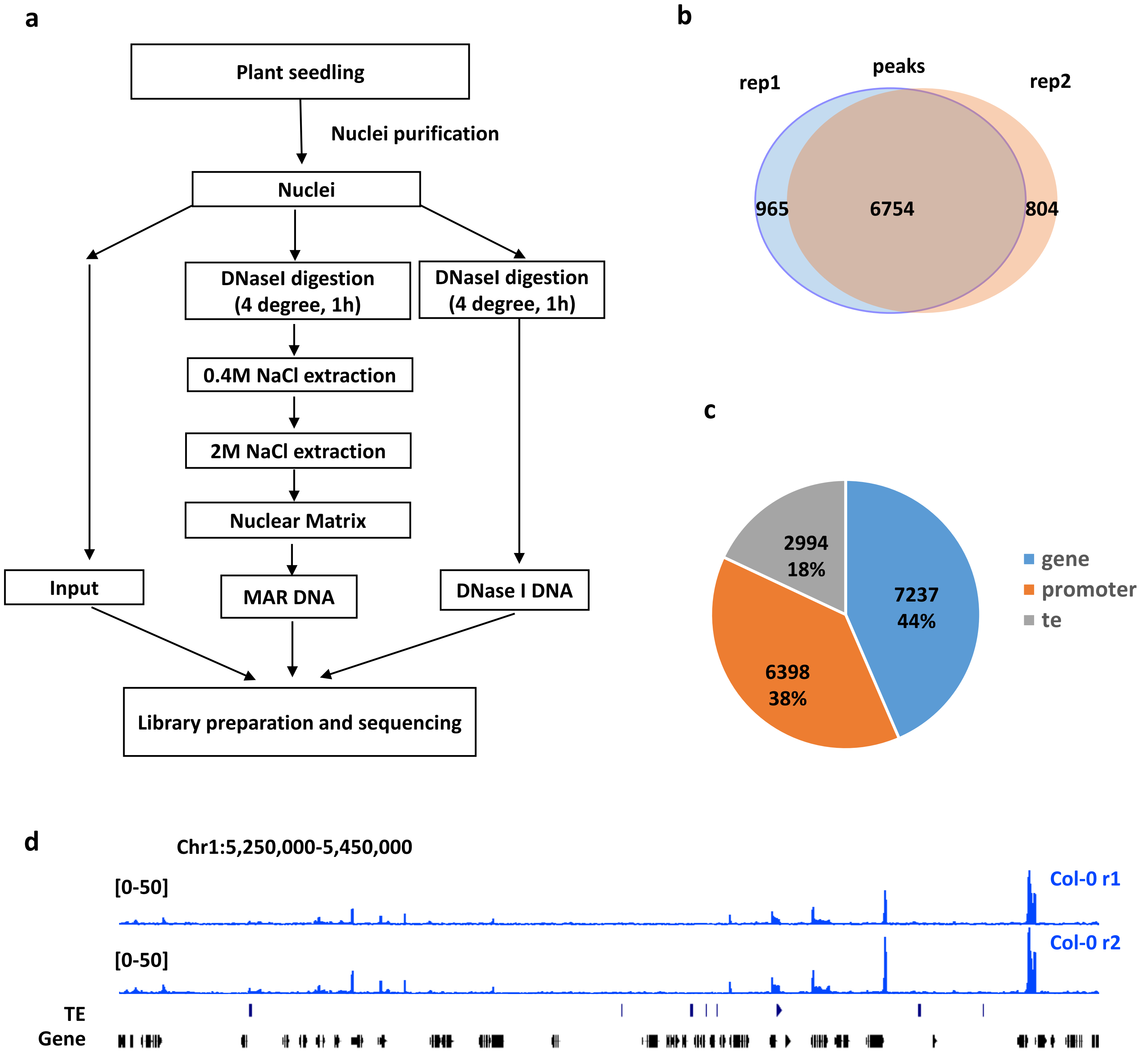

Supplementary Fig. 5. MAR-seq in WT and *ahl22 frs7 frs12*. (a) Flow diagram representing isolation of nuclear matrix and further DNA extraction. (b) Venn diagram showing the overlap of MAR peaks in WT between two replicates. (c) distribution of MAR peaks in genes, promoters, and TEs. (d) Genome browser views of a random selected region (Chr1: 5250000-5450000) showing MAR peaks in 2 replicates of Col-0. r1/r2 (replicate1/2).

Supplementary Fig. 6

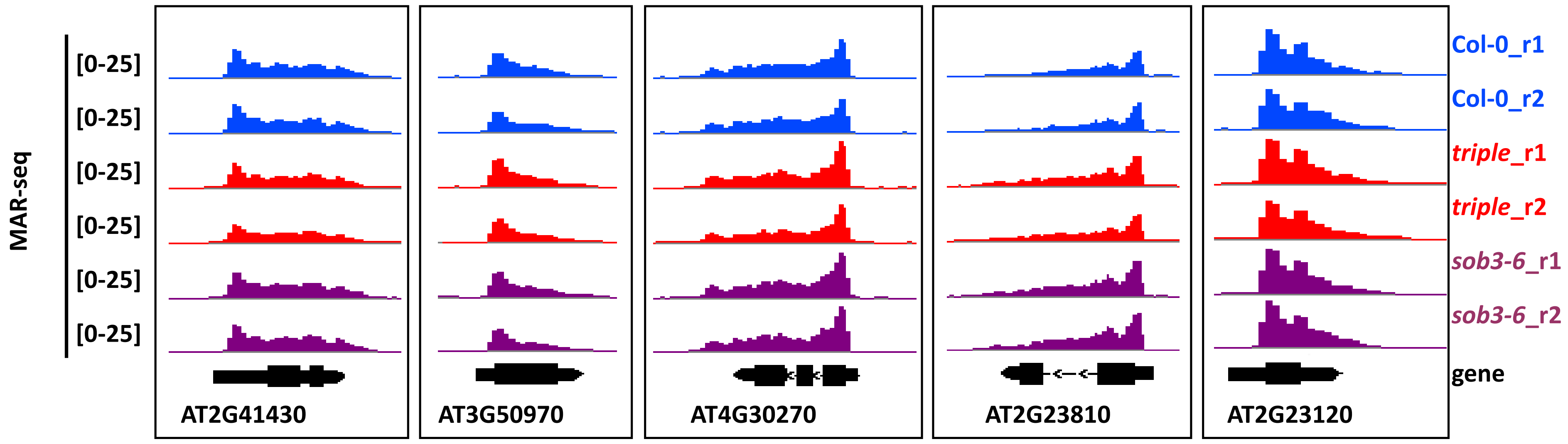

Supplementary Fig. 6. Genome browser views of two replicates of genes with no difference of MAR enrichment in Col-0, *triple* and *sob3-6*. r1/r2 (replicate1/2).

Supplementary Fig. 7

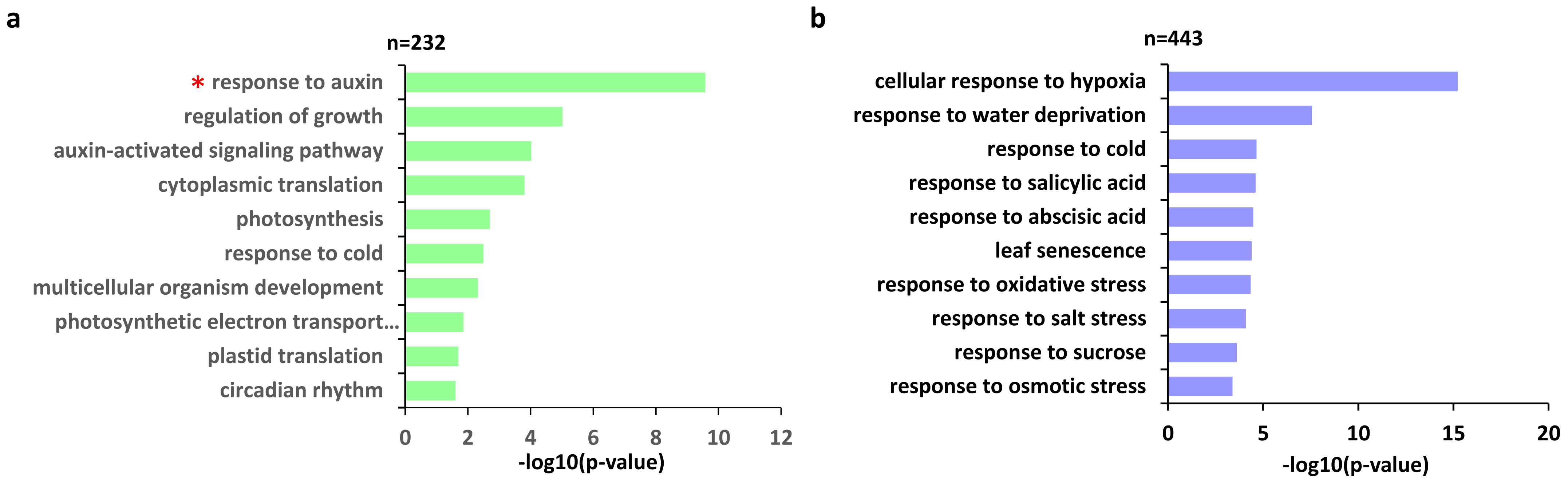

Supplementary Fig. 7. Gene ontology analysis of MAR decreased genes overlapped with DEGs in the triple mutant. (a, b). Enriched biological processes of common genes identified from MAR decreased genes and UGs (A, n=232) and DGs (B, n=443) in the triple mutant. The X-axis represent negative log10 (*p-value*).

Supplementary Fig. 8

a

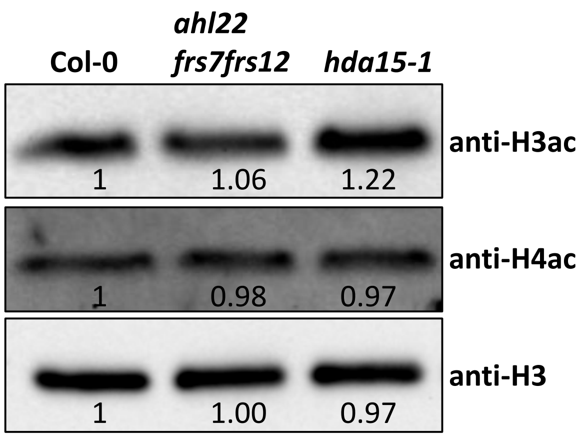

b

Accession	Gene ID	Gene	Coverage [%]	# Peptides
Q8GXJ1	AT3G18520	HDA15	31	14
O22446	AT4G38130	HDA19	43	12
Q8H0W2	AT3G44680	HDA9	17	7

Supplementary Fig. 8. Protein level analysis of histone acetylation and HDAs. (a) Western blot for H3ac, H4ac and H3 levels in total extracts from 5-d-old whole plants of Col-0, *ahl22 frs7 frs12* and *hda15-1*. (b) Identified histone deacetylases from the LC-MS analysis.

Supplementary Fig. 9

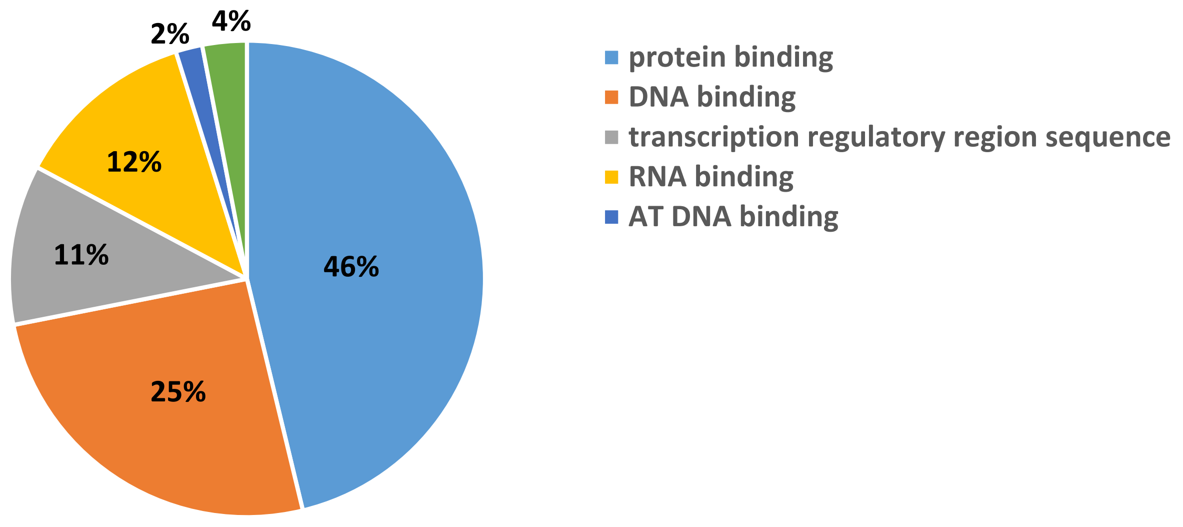

Supplementary Fig. 9. Enriched molecular function of nuclear matrix proteins from 5-d-old Col-0.

Supplementary Fig. 10

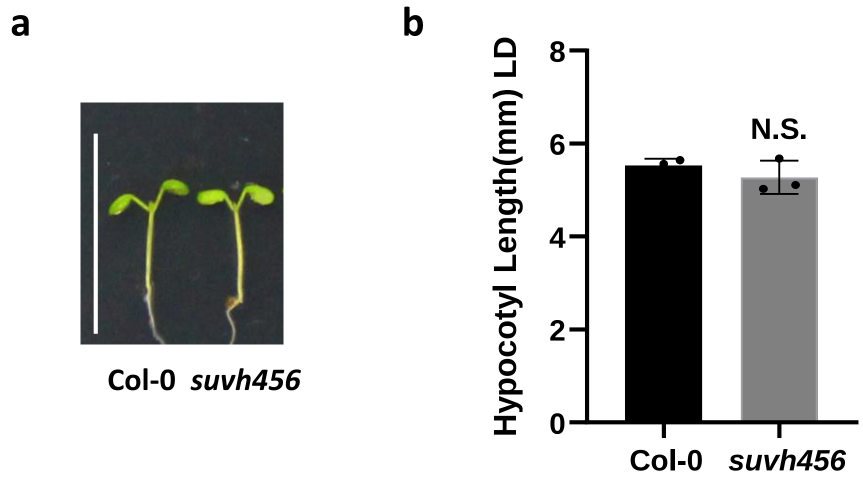

Supplementary Fig. 10. Hypocotyl phenotype of *suvh456*.

(a). The hypocotyl phenotype of indicated lines grown vertically under LD conditions (16h at 23 °C under 22 μmol·m^{-2}·s^{-1} continuous white light, 8h at 23 °C dark) for 5 days. Scale bar= 1 cm.

(b). Hypocotyl lengths of indicated lines grown under LD conditions (16h at 23 °C under 22 μmol·m^{-2}·s^{-1} continuous white light, 8h at 23 °C dark) for 5 days. Average length of three independent measurements ± standard deviations are shown. Each measurement with n=30 plants, 3 replicates in total. Unpaired two-tailed Student's t-test was used. N.S. *p value* >0.05, **p value* ≤ 0.05, ***p value* ≤ 0.01, ****p value* ≤ = 0.001, *****p value* ≤ = 0.0001.

Supplementary Fig. 11

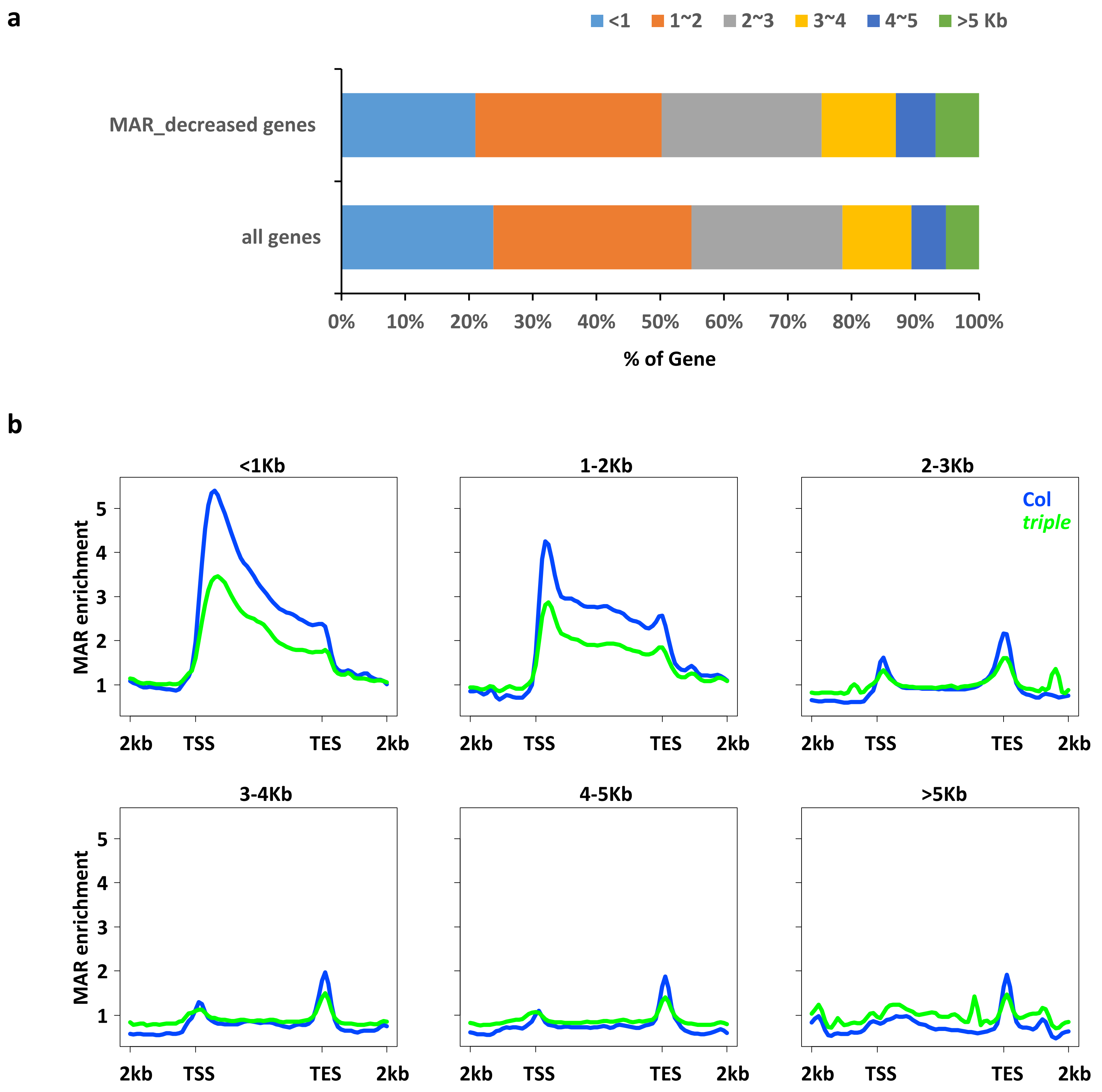

Supplementary Fig.11. Distribution of decreased MAR at different sizes of genes.
(a). Distribution of genes over gene size ranging from 2-3 kb, 3-4 kb, 4-5 kb and larger than 5 kb in MAR decreased genes and all genes.
(b). Metagene plot showing the average distribution of MAR enrichment over protein-coding genes grouped by gene size. TSS: transcription start site, TES: transcription end site.
