# Supplementary material for: Chromatin attachment to the nuclear matrix represses hypocotyl elongation in Arabidopsis thaliana
Source: Nat Commun. 2024 Feb 12;15:1286. doi: 10.1038/s41467-024-45577-5 (PMC10861482; doi:10.1038/s41467-024-45577-5)
Supplement: Supplementary file 3 — Description of Additional Supplementary Files [file 41467_2024_45577_MOESM3_ESM.pdf]

Description of Additional Supplementary Files:

Supplementary Data 1: TAP-MS analysis of AHL22.

Supplementary Data 2: LC-MS analysis of nuclear matrix proteins.

Supplementary Data 3: Differentially expressed genes in ahl22, frs7frs12, ahl22frs7frs12, and hda15- 1.

Supplementary Data 4: Common targets bound by SOB3 and FRS12.

Supplementary Data 5: MAR-seq of Col-0 and ahl22frs7frs12.

Supplementary Data 6: List of Primers Used in This Study.
