# Supplementary material for: Chromatin attachment to the nuclear matrix represses hypocotyl elongation in Arabidopsis thaliana
Source: Nat Commun. 2024 Feb 12;15:1286. doi: 10.1038/s41467-024-45577-5 (PMC10861482; doi:10.1038/s41467-024-45577-5)
Supplement: Supplementary file 10 — Reporting Summary [file 41467_2024_45577_MOESM10_ESM.pdf]

nature portfolio

Corresponding author(s): Hua Jiang

Last updated by author(s): Jan 16, 2024

Reporting Summary

Nature Portfolio wishes to improve the reproducibility of the work that we publish. This form provides structure for consistency and transparency in reporting. For further information on Nature Portfolio policies, see our Editorial Policies and the Editorial Policy Checklist.

Statistics

For all statistical analyses, confirm that the following items are present in the figure legend, table legend, main text, or Methods section.

n/a	Confirmed	
☐	☒	The exact sample size (*n*) for each experimental group/condition, given as a discrete number and unit of measurement
☐	☒	A statement on whether measurements were taken from distinct samples or whether the same sample was measured repeatedly
☐	☒	The statistical test(s) used AND whether they are one- or two-sided<br>*Only common tests should be described solely by name; describe more complex techniques in the Methods section.*
☒	☐	A description of all covariates tested
☒	☐	A description of any assumptions or corrections, such as tests of normality and adjustment for multiple comparisons
☐	☒	A full description of the statistical parameters including central tendency (e.g. means) or other basic estimates (e.g. regression coefficient) AND variation (e.g. standard deviation) or associated estimates of uncertainty (e.g. confidence intervals)
☐	☒	For null hypothesis testing, the test statistic (e.g. *F*, *t*, *r*) with confidence intervals, effect sizes, degrees of freedom and *P* value noted<br>*Give P values as exact values whenever suitable.*
☒	☐	For Bayesian analysis, information on the choice of priors and Markov chain Monte Carlo settings
☒	☐	For hierarchical and complex designs, identification of the appropriate level for tests and full reporting of outcomes
☒	☐	Estimates of effect sizes (e.g. Cohen's *d*, Pearson's *r*), indicating how they were calculated

Our web collection on statistics for biologists contains articles on many of the points above.

Software and code

Policy information about availability of computer code

Data collection	All sequencing samples generated in this study were prepared in house and sequenced on Illumina platforms.
Data analysis	HISAT 2 (v2.0.4)<br>Deseq 2 (v1.28.1)<br>FASTP (v0.20.0)<br>R (v4.1.0)<br>SCIER 2<br>Mascot Distiller (version 2.5.0, Matrix Science)<br>Mascot search engine (version 2.5.1, Matrix Science)<br>SymPhoTime software package (v5.2.4.0; PicoQuant)<br>GraphPad Prism (version 9.0.0)<br>Proteome Discoverer (version 2.4)<br>Sequest HT engine (Thermo Scientific)<br>ChemiDoc (BIO-RAD)<br>Detailed parameters are described in relative sections in "Methods".

For manuscripts utilizing custom algorithms or software that are central to the research but not yet described in published literature, software must be made available to editors and reviewers. We strongly encourage code deposition in a community repository (e.g. GitHub). See the Nature Portfolio guidelines for submitting code & software for further information.

Data

Policy information about availability of data

All manuscripts must include a data availability statement. This statement should provide the following information, where applicable:

- Accession codes, unique identifiers, or web links for publicly available datasets
- A description of any restrictions on data availability
- For clinical datasets or third party data, please ensure that the statement adheres to our policy

All sequencing data have been submitted to the NCBI Gene Expression Omnibus (GEO) under accession number GSE215135 (https://www.ncbi.nlm.nih.gov/geo/query/acc.cgi?acc=GSE215135). Proteome raw data has been deposited at MassIVE (https://massive.ucsd.edu/ProteoSAFe/dataset.jsp?task=aece25b2191440c8ac533865ee58545c) under the dataset ID MSV000090406.

Human research participants

Policy information about studies involving human research participants and Sex and Gender in Research.

Reporting on sex and gender	not applicable
Population characteristics	not applicable
Recruitment	not applicable
Ethics oversight	not applicable

Note that full information on the approval of the study protocol must also be provided in the manuscript.

Field-specific reporting

Please select the one below that is the best fit for your research. If you are not sure, read the appropriate sections before making your selection.

☒ Life sciences ☐ Behavioural & social sciences ☐ Ecological, evolutionary & environmental sciences

For a reference copy of the document with all sections, see nature.com/documents/nr-reporting-summary-flat.pdf

Life sciences study design

All studies must disclose on these points even when the disclosure is negative.

Sample size	No sample-size calculation was performed for each replicate. For MAR-seq, ChIP, and gene expression, the weight of each batch of harvested samples was approximately 1g, which was required as the starting material. For these experiments, such a sample size is widely accepted by the Arabidopsis research community.<br>Sample sizes of all box plots in this study were intrinsically linked to samples fulfilling a given selection criterion, which was described clearly in the manuscript.
Data exclusions	No data was excluded from the analyses.
Replication	ChIP-qPCR 2 biological replicates; MAR-seq data: 2 biological replicates; RNA-seq data: 2 biological replicates; RT-qPCR: 2 biological replicates; All the attempts at replication were successful.
Randomization	Plants with identical sample identity were grown in at least three pots or on three half-strength MS medium plates, which were placed randomly in the growth chamber.<br>For sample harvesting, randomization was also applied, in which control- and heat-stressed plants of the same genotype were randomly chosen from the corresponding pots or medium plates.
Blinding	Blinding was not applicable. For both the experiments in wet lab and NGS data analyses, we applied identical protocol and pipeline to individual samples, respectively.

Reporting for specific materials, systems and methods

We require information from authors about some types of materials, experimental systems and methods used in many studies. Here, indicate whether each material, system or method listed is relevant to your study. If you are not sure if a list item applies to your research, read the appropriate section before selecting a response.

Materials & experimental systems

n/a	Involved in the study
☐	☒ Antibodies
☒	☐ Eukaryotic cell lines
☒	☐ Palaeontology and archaeology
☒	☐ Animals and other organisms
☒	☐ Clinical data
☒	☐ Dual use research of concern

Methods

n/a	Involved in the study
☒	☐ ChIP-seq
☒	☐ Flow cytometry
☒	☐ MRI-based neuroimaging

Antibodies

Antibodies used

The following commercial antibodies were used: anti-GFP (B-2) antibody (SANTA CRUZ sc-9996, 1:50), anti-FLAG antibody (F1804, Sigma, 1:1000), anti-H3 (Sigma/H9289), anti-H3ac (Millipore /06-599), and anti-H4ac (Millipore/06-866).

Validation

All antibodies have been validated by the vendors. Primary antibodies that recognize HA- or GFP-tagged proteins were also validated by ourselves by including wild-type materials (for western blot) as negative controls.

Addition information from manufacturers:
anti-GFP (B-2) antibody (SANTA CRUZ /sc-9996)
https://www.scbt.com/p/gfp-antibody-b-2

anti-FLAG antibody (Sigma /F1804)
https://www.sigmaaldrich.com/DE/de/product/sigma/f1804

anti-H3 (Sigma /H9289)
https://www.sigmaaldrich.com/DE/de/product/sigma/h9289

anti-H3ac (Millipore /06-599)
https://www.merckmillipore.com/DE/de/product/Anti-acetyl-Histone-H3-Antibody,MM_NF-06-599?ReferrerURL=https%3A%2F%2Fwww.google.com%2F

anti-H4ac (Millipore/06-866)
https://www.merckmillipore.com/DE/de/product/Anti-acetyl-Histone-H4-Antibody,MM_NF-06-866?ReferrerURL=https%3A%2F%2Fwww.google.com%2F
